# Shunt configuration’s role in shaping hemodynamics of reverse Potts shunt in pediatric pulmonary arterial hypertension

**DOI:** 10.3389/fbioe.2025.1697468

**Published:** 2026-01-29

**Authors:** Jinlong Liu, Xiafeng Yu, Jiwen Xiong, Yi Yan, Yanjun Sun, Yumin Zhong, Hao Zhang

**Affiliations:** 1 Department of Cardiothoracic Surgery, Shanghai Children’s Medical Center, Shanghai Jiao Tong University School of Medicine, Shanghai, China; 2 Shanghai Institute for Pediatric Congenital Heart Disease, Shanghai Children’s Medical Center, Shanghai Jiao Tong University School of Medicine, Shanghai, China; 3 Institute of Pediatric Translational Medicine, Shanghai Children’s Medical Center, Shanghai Jiao Tong University School of Medicine, Shanghai, China; 4 Shanghai Engineering Research Center of Virtual Reality of Structural Heart Disease, Shanghai Children’s Medical Center, Shanghai Jiao Tong University School of Medicine, Shanghai, China; 5 Department of Radiology, Shanghai Children’s Medical Center, Shanghai Jiao Tong University School of Medicine, Shanghai, China

**Keywords:** computational fluid dynamics, hemodynamics, pulmonary arterial hypertension, reverse Potts shunt, virtual design

## Abstract

**Objectives:**

Reverse Potts shunt is a promising yet high-risk therapy for pediatric pulmonary arterial hypertension. Postoperative hemodynamics is critically influenced by shunt configuration but is difficult to predict. This study aimed to quantify the effects of shunt size and location on hemodynamics to guide surgical planning.

**Methods:**

Based on a patient-specific model, four postoperative models with two different shunt locations [left pulmonary artery (LPA)–descending aorta (DAO) and pulmonary artery bifurcation–aortic arch] and three conduit sizes (4, 5, and 6 mm) were created. The direct Potts shunt model was created by a direct side-to-side anastomosis between the LPA and DAO with a 6-mm circular opening. Quantitative parameters including the shunt ratio (SR), which was defined as the percentage of the shunt flow rates to the total pulmonary inflow rate, lower limb oxygen saturation, and pressure were analyzed.

**Results:**

Increasing the shunt size from 4 mm to 6 mm elevated the SR from 6.01% to 9.80%, concurrently reducing lower limb oxygen saturation from 89.57% to 86.52%. When taking 11,000 Pa as the threshold, this increased SR resulted in a reduction of the high-pressure area from 17.32% of the total pulmonary artery area to almost zero. Meanwhile, the high-pressure area on the aorta expanded from 8.72% of the total aortic area to 14.94%. These results indicated a reduction in the right ventricular afterload and an increase in the left ventricular afterload. Notably, a 6-mm shunt at the pulmonary artery bifurcation yielded a significantly larger SR than at the LPA (9.80% vs. 2.68%), which is attributed to a higher pressure gradient at the pulmonary artery bifurcation (1,201 Pa vs. 162 Pa).

**Conclusion:**

The shunt location had a greater impact on the SR than shunt size within the 4 mm–6 mm range in this specific case. A 6-mm shunt at the pulmonary artery bifurcation yielded a significantly larger SR than at the LPA, which is attributed to the higher preoperative pressure gradient at the bifurcation site. Left heart function is as critical as right heart function in maintaining pressure balance and determining outcomes, as the shunt flow increases the left ventricular afterload.

## 1 Introduction

Pulmonary arterial hypertension (PAH) is a progressive disease characterized by elevated pressure in the pulmonary artery, resulting in right heart failure and eventually death (Ivy et al., 2013). The median survival time of untreated PAH patients is only 2.8 years (D'Alonzo et al., 1991). Inspired by the long-term survival of patients with Eisenmenger syndrome, reverse Potts shunt was applied to treat patients with PAH by mimicking the right-to-left shunting (Cantor et al., 1999; Blanc et al., 2004; Rosenzweig et al., 2019; Diller et al., 2006). Reverse Potts shunt is performed by a direct side-to-side anastomosis, or conduit implantation, or trans-catheter stent implantation between the left pulmonary artery (LPA) and the descending aorta (DAO), thereby effectively decreasing the pulmonary arterial pressure and alleviating right ventricular failure (Rosenzweig et al., 2021; Esch et al., 2013). Several studies have reported promising results of using reverse Potts shunt with prolonged survival and long-lasting improvement in functional capacities in pediatric PAH patients (Esch et al., 2013; Baruteau et al., 2012; Baruteau et al., 2015; Lancaster et al., 2021; Gorbachevsky et al., 2017).

**FIGURE 1:**
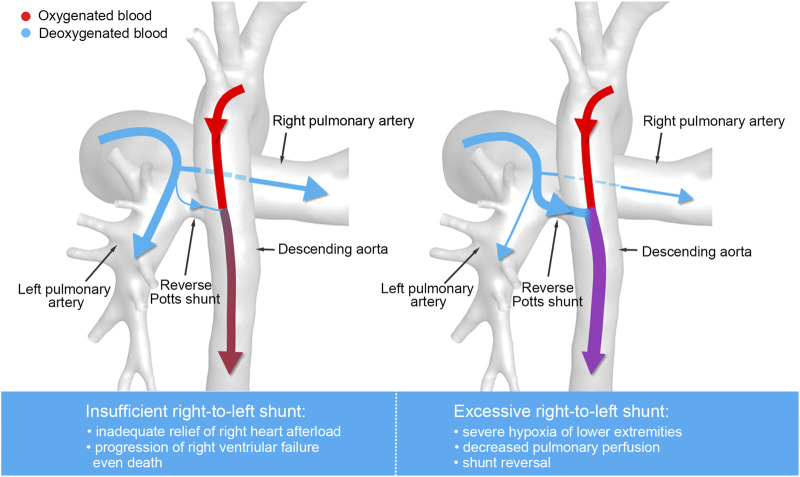
Illustration of the blood flow distribution of reverse Potts shunt.

Despite advances in surgical techniques and management, use of reverse Potts shunt is still associated with high mortality of up to 15% (Grady et al., 2021) because postoperative hemodynamic changes are difficult to estimate, especially the control of blood flow balance between pulmonary circulation and systemic circulation, resulting in difficulties in surgical planning and perioperative managements. As shown in Figure 1, severe right-to-left shunting results in decreased pulmonary perfusion, severe hypoxia of lower extremities, and even shunt reversal. Conversely, the insufficient right-to-left shunting may lead to progression of right ventricular failure, exercise intolerance, and eventually death. The postoperative hemodynamics was affected by physiological factors including cardiac output, vascular resistance, and vascular morphology and by the surgical design, including the shunt size and the shunt location, which are key factors that surgeons pay attention to during preoperational planning. However, the effects of these factors on postoperative hemodynamics are less investigated and there is no definite criterion regarding the reverse Potts shunt design.

Computational fluid dynamics (CFD) and computer-aided design (CAD) are effective tools for individualized surgical design and hemodynamic evaluation of congenital heart diseases (Xiong et al., 2021; Wang et al., 2020; Sun et al., 2014; Whitehead et al., 2007). Delhaas T and colleagues using the lumped parameter model (CircAdapt) reported pressure and oxygen saturation following reverse Potts shunt with different shunt sizes. The lumped parameter model is a one-dimensional model that lacked the consideration of the vascular morphology (Delhaas et al., 2018). Pant et al. (2022) presented a multiscale model with PAH physiology and investigated hemodynamic parameters after the trans-catheterized reverse Potts shunt with various shunt sizes between the LPA and the DAO. However, the hemodynamic performances of different surgical methods and shunt locations were less studied.

The present study aimed for a better understanding of postoperative hemodynamics based on patient-specific PAH physiology and different surgical designs of reverse Potts shunt using CFD and CAD. Flow features and hemodynamic parameters including the shunt ratio (SR), pressure, wall shear stress (WSS) and oxygen saturation were assessed to evaluate the suitability of the reverse Potts shunt design in the specific PAH patient.

## 2 Materials and methods

### 2.1 Clinical data acquirement

A 3-year-old boy diagnosed with severe PAH was included in this study. The local institutional review board and regional research ethics committee of Shanghai Children’s Medical Center (SCMC), Shanghai Jiao Tong University School of Medicine, approved this study (Approval No.SCMCIRB-K2023023-1 on 24 February 2023), and informed consent was obtained from the patient’s parents. The patient-specific enhanced chest computed tomography (CT) images were acquired using 64-slice spiral CT scanner (GE Discovery CT750 HD, America) before surgery. The peak velocity and average flow rates through the ascending aorta (AAO) and the main pulmonary artery (MPA) were obtained by echocardiography with real-time electrocardiogram simultaneously. Preoperative catheterization was applied to measure the blood pressure at the DAO and the MPA. The patient-specific clinical data before surgery are listed in Table 1.

**TABLE 1:** Patient-specific clinical data before surgery. The average flow rate and peak velocity through the ascending aorta (AAO) and the main pulmonary artery (MPA) were obtained by echocardiography. The blood pressures of the MPA and the descending aorta (DAO) were measured by cardiac catheterization.

Position	Average flow rate (mL/s)	Systolic peak velocity (m/s)	Systolic pressure (mmHg)	Diastolic pressure (mmHg)	Mean pressure (mmHg)
MPA	40.04	0.61	88	42	65
AAO	33.58	1.00	—	—	—
DAO	—	—	84	44	62

### 2.2 3D model reconstruction and virtual design

**FIGURE 2:**
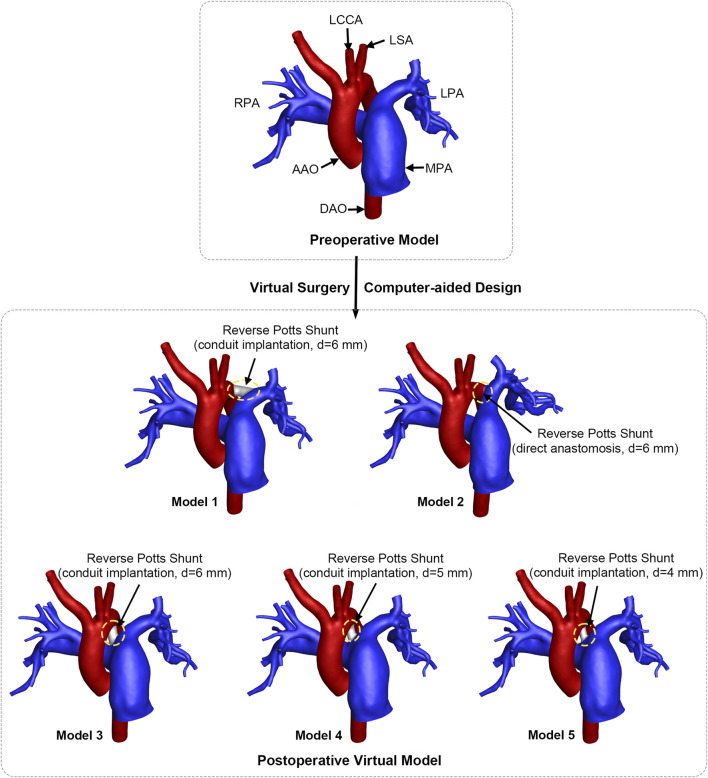
Geometrical models and virtual surgery design of reverse Potts shunt. RPA, right pulmonary artery; LPA, left pulmonary artery; MPA, main pulmonary artery; AAO, ascending aorta; DAO, descending aorta; LCCA, left common carotid artery; LSA, left subclavian artery; d, diameter. The circle with yellow dashed line indicates the shunt location.

The preoperative vascular model was reconstructed by Materialise®-Mimics Innovation Suite 20.0 (Materialise NV. Leuven, Belgium) based on individualized chest CT images. Pulmonary artery branches were preserved as much as possible to ensure the authenticity of the vascular model. In addition, the aorta and three aortic branches were reconstructed. Non-shrinking smoothing was applied to generate the numerical model for CFD analysis. The geometry of the preoperative vascular model is depicted in Figure 2.

Virtual surgeries were performed and five postoperative virtual models were created using the CAD technique, as shown in Figure 2. According to the surgeon’s recommendation, the conduit diameter should be approximately 70% of the diameter of DAO. A 6-mm conduit was selected and implanted between the LPA and the DAO, and the postoperative model was named Model 1. Model 2 was created to simulate a direct side-to-side LPA–DAO anastomosis with a 6-mm circular opening. The LPA and DAO were pulled together to form a contact area, and then, a 6-mm circular opening was created. Thus, the angle between the LPA and the right pulmonary artery (RPA) in Model 2 was smaller than that in the preoperative model (93.28° vs. 108.47°). The aortic arch deviated to the left side. To include all possible surgical designs, the 6-mm conduit was implanted at the site of the original ductus arteriosus between the aortic arch and pulmonary artery bifurcation, and it was named Model 3. Two other conduits of different sizes (5 mm and 4 mm) were interpolated to explore the impacts of shunt size, and these virtual models were named Model 4 and Model 5 in sequence.

### 2.3 Numerical simulation

#### 2.3.1 Mesh generation

All models were discretized via ANSYS®-ICEM CFD 2020 (ANSYS Inc., America) for calculation. The tetrahedral mesh was fabricated in the interior, and five layers of prism mesh were generated along the vascular wall. Grid-independent verification was performed, and the wall shear stress was set as the indicator, with the quantity of grids changing. The results indicated that a grid number of approximately two million would generate the most efficient mesh. Detailed mesh information of all the models is shown in Table 2, and this mesh is sufficient to produce robust calculations.

**TABLE 2:** Mesh information of all models.

Model	Total nodes	Total elements
Preoperative model	490,495	2,699,564
Model 1	494,766	2,723,702
Model 2	489,382	2,692,974
Model 3	492,438	2,710,545
Model 4	492,010	2,708,529
Model 5	491,314	2,704,234

#### 2.3.2 Boundary conditions

The AAO and the MPA were inlets. The inflow was the systolic peak velocity, and the average flow rate was measured by echocardiography, as listed in Table 1. The DAO, aortic branches, and the bilateral pulmonary artery branches were the pressure outlets. For the simulation of the preoperative model, the outlet pressure was assumed according to the systolic pressure measured by the cardiac catheterization, as well as the average arterial pressure. The reverse Potts shunt is designed to reduce the elevated pulmonary pressure to the level of systemic pressure (Baruteau et al., 2015). In other words, the pressure equilibrium between the pulmonary circulation and systemic circulation is a key physiological feature after shunt establishment. Therefore, in order to predict the hemodynamics after reverse Potts shunt, the same pressure conditions should be set at all pressure outlets. For postoperative models, the reference pressure of 65 mmHg was imposed on all the outlets for simulation of the systolic peak. The reference pressure of 60 mmHg was imposed on all the outlets for simulation of the average flow rate. Both the vascular wall and conduit wall were assumed as rigid and impermeable.

#### 2.3.3 Simulations

Twelve states of steady simulations were carried out using ANSYS®-Fluent 2020 (ANSYS Inc., America). Blood was simplified as a Newtonian fluid with a constant density of 1,060 kg·m^-3^ and viscosity of 0.0035 Pa·s, because of high-speed and high-shear blood flow in large arteries and because the diameter of great vessels is much larger than the diameter of a single red blood cell (Liu et al., 2015; Ceballos et al., 2019). The semi-implicit (SIMPLE) method and first-order upwind scheme were applied. The convergence criterion was 10^−5^.

#### 2.3.4 Hemodynamic parameters

To quantitatively evaluate the hemodynamic characteristics of reverse Potts shunt, the SR was defined as the percentage of the shunt flow rates to the total MPA inflow rate. The shunt flow rate was calculated by determining the deviation between the inflow rate of MPA and the outflow rates of all the pulmonary artery branches. The outflow rates of all the pulmonary artery branches were calculated as the sum of the LPA and RPA outflow rates, which could be obtained from the simulation results. Therefore, the SR could be calculated according to Equation 1:

$$SR=\frac{Q_{MPA}-Q_{P}}{Q_{MPA}}\times 100\%=\frac{Q_{MPA}-\left(Q_{LPA}+Q_{RPA}\right)}{Q_{MPA}}\times 100\%,$$ (1)

where $Q_{MPA}$ is the inflow rate of the MPA, $Q_{P}$ is the sum of the outflow rates of the LPA and RPA, $Q_{LPA}$ is the outflow rate of the LPA, and $Q_{RPA}$ is the outflow rate of the RPA.

The pulmonary-to-systemic ratio (*Q*_*P*_ */Q*_*S*_ *)* and the LPA/RPA ratio (*Q*_*LPA*_ */Q*_*RPA*_) were calculated to evaluate the flow distribution as well. The *Q*_*S*_ is the sum of the outflow rates of the DAO and aortic branches.

The lower limb oxygen saturation could be estimated by the oxygen saturation of the mixed blood in DAO. In a reverse Potts shunt, part of deoxygenated blood from the MPA was shunted into the DAO, where it mixed with oxygenated blood from the AAO. The shunt flow from the MPA is calculated as (Q*_MPA_*‐Q*_P_*), and the oxygenated blood flow from the AAO equaled to the DAO flow minus shunt flow. The oxygen saturation was assumed to be 95% in the AAO and 65% in the MPA. The dissolved oxygen saturation was ignored. The oxygen saturation of the mixed blood of DAO could be calculated using Equation 2:

$$S_{m}O_{2}=\frac{\left(\left(Q_{MPA}-Q_{P}\right)\times 65\%+\left(Q_{DAO}-\left(Q_{MPA}-Q_{P}\right)\right)\times 95\%\right)}{Q_{DAO}},$$ (2)

where $Q_{DAO}$ is the blood outflow rate from the DAO.

WSS is related to vascular endothelial injuries and thrombosis (Kumar et al., 2018; Koskinas et al., 2012), and it is calculated based on Equation 3:

$$\tau_{w}={-\mu \left.\frac{\partial u_{x}}{\partial y}\right|}_{y=0},$$ (3)

where *μ* is the fluid viscosity, $u_{x}$ is the velocity of fluid near the vessel wall and y is the distance to the vessel wall.

## 3 Results

### 3.1 Flow distribution and oxygen saturation

The largest shunt flow was observed in Model 3 which occupied 9.80% of MPA inflow, as listed in Table 3. Meanwhile, the oxygen saturation at the DAO and the pulmonary-to-systemic ratio were the lowest in Model 3 since the largest amount of deoxygenated pulmonary flow was shunted into the DAO. The shunt flow of Model 1 and Model 2 was similar, indicating that the surgery methods did not change the postoperative flow distribution considerably. The shunt flow ratio decreased from 9.80% to 6.01%, and the oxygen saturation at the DAO increased with decreasing shunt size. The shunt flow of Model 5 is more than that of Model 1. It appeared that the shunt flow was more prone to be affected by its location instead of size in this case. The pulmonary-to-systemic ratio reflected the flow distribution between the pulmonary circulation and systemic circulation after the reverse Potts shunt, and it changed following the shunt ratio. The LPA/RPA ratio barely changed with the change in the shunt location and size.

**TABLE 3:** Flow distribution and oxygen saturation in the descending aorta (DAO). Q_P_, Q_S_, Q_LPA_, and Q_RPA_ are flow rates to pulmonary circulation, systemic circulation, left pulmonary artery, and right pulmonary artery, respectively.

Model	Shunt ratio (%)	Oxygen saturation in the DAO (%)	Q_P_/Q_S_	Q_LPA_/Q_RPA_
Preoperative model	—	—	1.19	1.00
Model 1	2.68	92.50	1.12	0.99
Model 2	2.52	92.60	1.13	0.99
Model 3	9.80	86.52	0.96	1.00
Model 4	8.26	87.74	1.00	0.99
Model 5	6.01	89.57	1.05	0.99

### 3.2 Pressure

**FIGURE 3:**
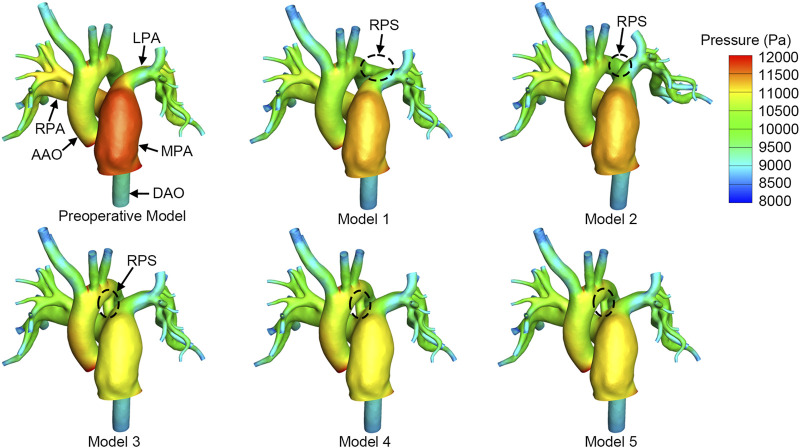
Pressure distribution at the systolic peak. The circle with black dashed line indicates the reverse Potts shunt (RPS). MPA, main pulmonary artery; AAO, ascending aorta; LPA, left pulmonary artery; RPA, right pulmonary artery; DAO, descending aorta.

Since the shunt flow directly leads to the decrease in MPA pressure, the higher the shunt flow, the lower the pressure in the MPA. As shown in Figure 3, Model 1 and Model 2 exhibited higher MPA pressure of approximately 11,300 Pa. Model 3 had the lowest MPA pressure, which was 350 Pa lower than that of Model 2, because there was increased pulmonary flow shunting into the DAO in Model 3. As the shunt size increased from 4 mm to 6 mm, the MPA pressure gradually decreased by 160 Pa, while the AAO pressure increased by 190 Pa. Although the numerical changes are not substantial, the high-pressure region varied significantly. With 11,000 Pa as the threshold, the high-pressure area on the aorta expanded with the increase in the shunt size, from 4.36 cm^2^ to 7.47 cm^2^, accounting for 8.72%–14.94% of the total aortic area. In contrast, the high-pressure region in the MPA gradually decreased from 18.01 cm^2^ to 0.33 cm^2^, which is equivalent to a reduction in the high-pressure area from 17.32% of the total pulmonary artery area to almost zero.

### 3.3 Velocity streamline

**FIGURE 4:**
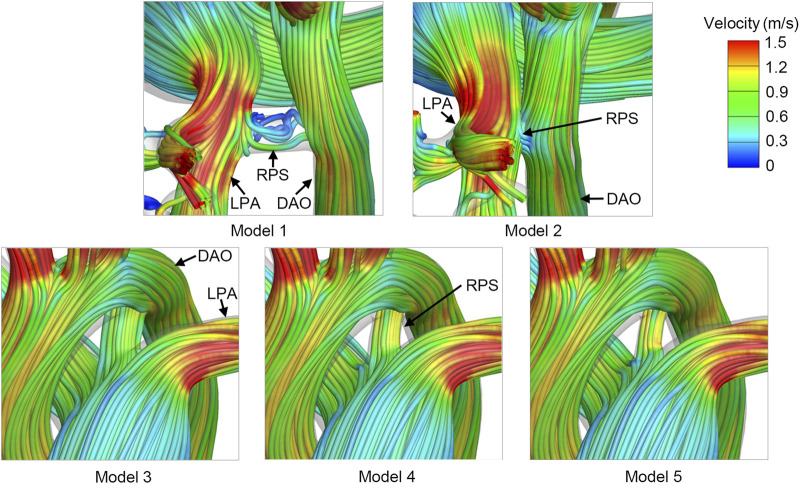
Velocity streamlines at the systolic peak of postoperative models. RPS, reverse Potts shunt; LPA, left pulmonary artery; DAO, descending aorta.

The flow vortex and flow stagnation with low velocity was observed in the reverse Potts shunt of Model 1, shown in Figure 4. The velocity streamlines were smoother and faster in reverse Potts shunt of Model 3, Model 4, and Model 5. In addition, with the decrease in the shunt size, the velocity through the shunt increased. Only a few streamlines passed through the reverse Pott shunt of Model 2, which could hardly be displayed in post-processing, indicating a lower shunt flow rate.

### 3.4 Wall shear stress

**FIGURE 5:**
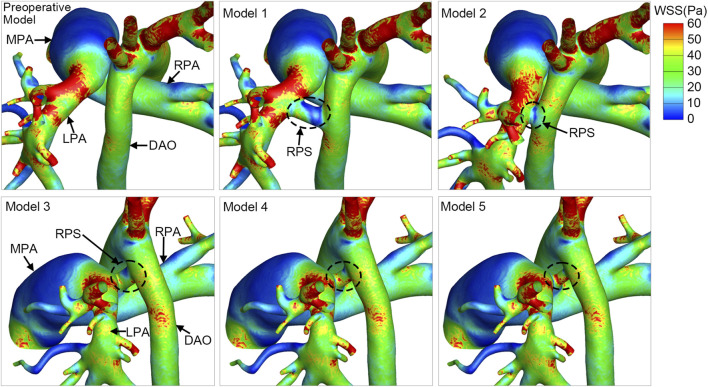
The distribution of wall shear stress (WSS) at the systolic peak. The circle with black dashed line indicates the reverse Potts shunt (RPS). MPA, main pulmonary artery; DAO, descending aorta; LPA, left pulmonary artery; RPA, right pulmonary artery.

The WSS was extremely low in the MPA of all the models, which was a combined result of the dilation of the MPA lumen and the relatively slow flow, as depicted in Figure 5. In addition, the WSS was high at the beginning of the LPA, resulting from the rapid flow through the narrowed site in LPA, compared with the expanded lumen of the MPA. The difference in the WSS distribution was insignificant in the pulmonary artery and the aorta among all the models, except for the WSS in the shunt. The shunt of Model 1 had extremely low WSS. On the contrary, the shunt was associated with higher WSS in Model 3, Model 4 and Model 5. The WSS slightly increased as the shunt size decreased from 6 mm to 4 mm.

## 4 Discussion

Reverse Potts shunt has been an emerging therapeutic strategy for pediatric PAH since a decade ago and is now used for some patients with severe cases without the exclusion of future transplantation. Although it has been applied in many countries, it is still in the primary stage and is associated with high risk and mortality. More evidence is required to support clinical decision-making including surgical planning and perioperative and postoperative management. We investigated hemodynamic characteristics of different surgical designs based on a patient-specific anatomy and physiological conditions through CAD and CFD. The shunt flow and oxygen saturation were quantitatively evaluated while changing the shunt size and shunt location, as well as using different surgical methods, which contribute to clinical management and improvement in reverse Potts shunt in pediatric PAH patients.

A certain right-to-left shunting range to reduce pulmonary arterial pressure is crucial to the prognosis of reverse Potts shunt. The shunt volume cannot be too small to reduce pulmonary artery pressure and right ventricle workload. In addition, it cannot be too high since excessive shunt flow will lead to extreme hypoxia of the lower limbs. However, the lowest shunt volume to allow for alleviation and the highest shunt volume to avoid extreme hypoxia of the lower limbs remain unknown. According to clinical experiences from our institute, the shunt flow was considered acceptable when the oxygen saturation in the lower limbs ranged from 80% to 85% after reverse Potts shunt. This statement is consistent with those of previous studies (Lancaster et al., 2021; Bobhate et al., 2021), which state that the oxygen saturation of the lower extremity should be 10%∼20% lower than that of the upper extremity. We estimated that the oxygen saturation of the lower limbs was within 86.52% to 92.60% after surgery. Corresponding with the oxygen saturation, the shunt ratio and the pulmonary-to-systemic ratio are shown in Table 3. Because there are currently no effective measures to evaluate shunt flow in clinical practice, the quantitative relationship revealed in this study will help surgeons evaluate the flow distribution and surgical effects in the clinical scenario.

The choice of the shunt location and shunt size has posed challenges for surgeons in preoperative planning. The position between the LPA and the DAO is generally selected, and the original ductus arteriosus may also be considered for stent implantation. Baruteau AE et al. (2015) mentioned that the choice of the shunt size should meet the purpose of pressure equilibrium between the pulmonary circulation and systemic circulation, and no accurate criterion was given. Lancaster et al. (2021) reported that 80%∼90% of the diameter of the DAO was selected in order to equalize the pulmonary pressure and systemic pressure, irrespective of whether conduit implantation or direct anastomosis was applied. In the present study, given the pressure difference between the MPA and DAO, 50%∼70% of the diameter of the DAO was selected, and the oxygen saturation for all the models was in an acceptable range. In fact, for a closed-loop flow system, pressure equilibrium will eventually be achieved irrespective of the shunt size and shunt location chosen. The key point is the time needed and the body’s tolerance in the process of achieving equilibrium. In addition, notably, the change in the SR was greater when the shunt was moved from the LPA (Model 1) to the pulmonary artery bifurcation (Model 3). This was because the pressure gradient between the pulmonary artery bifurcation and aortic arch was higher than the pressure gradient between the LPA and DAO (1,201 Pa vs. 162 Pa), which could be found in the preoperative model (Figure 3). It is indicated that the pressure difference also played an important role in shunt flow control. This point was similar with that of Gorbachevsky et al. (2017). They believed that the pulmonary artery-to-aorta mean pressure ratio is also critical to the choice of shunt size. If the pulmonary artery-to-aorta mean pressure ratio is above 1.5, the cross-sectional area of the shunt should be less than 0.4 of the cross-sectional area of DAO, which is approximately 62% of the diameter of the DAO. This is because the smaller shunt size will limit the shunt flow and allow for adjustment in postoperative management when the patient is vulnerable. Thus, the shunt size and shunt location should be carefully chosen based on the patient’s physiological conditions, especially the pressure gradient across the intended shunt site.

Postoperative pulmonary artery pressure and aortic pressure are important manifestations of cardiac afterload. The MPA pressure could also be extended to assess the effects of reverse Potts shunt on PAH treatment, since the decrease in MPA pressure is the direct result of shunt volume. The more the pulmonary flow was shunted, the lower MPA pressure and the higher AAO pressure would be, indicating the increase in left ventricular afterload. Although the numerical changes are not substantial in the present study, the high-pressure region experienced significant changes. When taking 11,000 Pa as the threshold, the increased shunt size led to a reduction in the high-pressure area from 17.32% of the total pulmonary artery area to almost zero. Meanwhile, the high-pressure area on the aorta expanded from 8.72% of the total aortic area to 14.94% with the increase in the shunt size. An increase in the high-pressure region will increase flow resistance, thereby imposing additional workload on the heart. These results indicated a reduction in right ventricular afterload and an increase in left ventricular afterload. The excessive shunt flow also leads to the reduction in pulmonary venous return to the left ventricle, indicating the decrease in left ventricular preload. As previously pointed out, a closed-loop system will ultimately reach a state of pressure equilibrium. The critical issue is physiological tolerance the functional status of the left and right ventricles and their ability to withstand the changes in preload and afterload caused by the shunt are one of the most important criteria for evaluating physiological tolerance. Initially, surgeons in our hospital concentrated mainly on right heart tolerance. With growing experience, the equal importance of the left heart function was established, and excessive shunt flow was identified as harmful due to the substantial load it places on the left heart. Boudjemline Y. et al. also reported that two cases of preoperative left heart function impairment had low cardiac output syndrome due to excessive shunt flow after the operation (Boudjemline et al., 2017). Thus, the left cardiac function should be carefully evaluated during the peri- and post-operative period.

WSS reflects the interaction between the vascular wall and blood flow, which could be sensed by the vascular endothelium, triggering a series of adaptive structural and functional changes (Kumar et al., 2018; Koskinas et al., 2012). The main difference in the WSS distribution was presented in the reverse Potts shunt. Low WSS was found in the shunt located between the LPA and the DAO, which could be explained by the flow vortex and flow stagnation. Although the shunt volume of Model 1 could be accepted, the flow vortex will increased the risk of thrombosis and shunt occlusion.

There were several limitations in this study. First, the elasticity and compliance of the vascular wall were not considered. Although the fluid–structure interaction could be applied to consider the properties of vascular wall and conduit, the calculation could be complicated and time-consuming. Second, due to the computational practicality and parameter uncertainty, multiphase modeling was not used to evaluate oxygen transportation. Using a simplified method focusing on the mixing of blood streams with different oxygen saturations was clinically considered acceptable and efficient. Third, the sample size was small, and only one case of a PAH patient was included, which may limit the application of the results. Specifically, the pulmonary-to-systemic pressure ratio was approximately 1.0 in this case, and the results may differ substantially for patients with a pulmonary-to-systemic pressure ratio significantly greater or less than 1.0. Nevertheless, the present study focused on the individualized surgical planning of reverse Potts shunt by introducing CAD and CFD. Notably, surgical planning for each patient is critical because each patient has only one chance. Future work investigating the effects of specific vascular anatomy and pulmonary-to-systemic pressure ratio on hemodynamic characteristics is needed to provide more generalizable guidelines. Finally, trans-catheter Potts shunt (TPS) has become an important method for treating severe PAH (Haddad et al., 2022; Anderson, et al., 2022; Boudjemline, et al., 2017). Although there are similarities between the trans-catheter and surgical methods, in part, the flow features of TPS differ from those of surgical Potts shunt due to stent protrusion and malformation. The hemodynamics of TPS requires further investigation considering the stent protrusion and malformation.

## 5 Conclusion

In conclusion, our patient-specific CFD analysis quantified the hemodynamic effects of reverse Potts shunt based on different shunt configurations. The key finding is that the shunt location had a greater impact on the SR than the shunt size within the 4 mm–6 mm range in this specific case. A 6-mm shunt at the pulmonary artery bifurcation yielded a significantly larger SR than at the LPA, which is attributed to the higher preoperative pressure gradient at the bifurcation site. Furthermore, our data underscore that the left ventricular function is as critical as the right ventricular function in determining the hemodynamic outcomes as the shunt flow increases left ventricular afterload. CAD and CFD facilitate a deep understanding of hemodynamic characteristics and are promising tools in helping individualized surgical design and postoperative prediction for these high-risk procedures.

## Data Availability

The original contributions presented in the study are included in the article/supplementary material; further inquiries can be directed to the corresponding author.
